# An experimental investigation of the stigmatization of weight loss and regain from GLP-1 receptor agonist use and cessation

**DOI:** 10.1038/s41366-026-02061-y

**Published:** 2026-04-03

**Authors:** Erin C. Standen, Sean M. Phelan, A. Janet Tomiyama

**Affiliations:** 1 Department of Psychological Sciences, Rice University, Houston, TX USA; 2 Robert D. and Patricia E. Kern Center for the Science of Health Care Delivery, Mayo Clinic, Rochester, MN USA; 3 Division of Health Care Delivery Research, Mayo Clinic, Rochester, MN USA; 4 Department of Psychology, University of California, Los Angeles, Los Angeles, CA USA

**Keywords:** Weight management, Public health, Risk factors

## Abstract

**Background/Objectives:**

Glucagon-like peptide-1 receptor agonist medications (GLP-1s) are effective for weight loss, but when people discontinue them, they tend to regain weight. The present work sought to examine the stigma of losing and regaining weight after GLP-1 use and cessation.

**Subjects/Methods:**

In two randomized, between-subjects experiments, participants evaluated a fictional target after reading a brief description of the target’s weight-related history, which varied by study condition. Study 1 (*N* = 607) aimed to understand stigma directed at individuals who lose weight using GLP-1s, so the target was described as either: having lost weight by using a GLP-1, having lost weight via diet and exercise, or not having lost weight. Study 2 (*N* = 706) examined stigma directed at people who regain weight after discontinuing GLP-1s, and the target was described as either: having regained weight after discontinuing a GLP-1, having regained weight after discontinuing a diet and exercise plan, not having lost weight, or having maintained weight loss. After reading, participants completed measures of weight-related stereotyping and willingness to affiliate with the target.

**Results:**

In Study 1, the GLP-1 target was evaluated more negatively than the diet/exercise target and the no weight loss target. Participants’ willingness to affiliate differed significantly across groups (*p* < 0.001), with the GLP-1 target receiving significantly lower ratings compared to the diet/exercise target (Mean difference = 0.52, 95% CI [0.27, 0.76]) and the no weight loss target (Mean difference = 0.26, 95% CI [0.02, 0.51]). In Study 2, the GLP-1 and diet/exercise targets were evaluated similarly, and both were rated more negatively than the target who maintained weight loss (*p*s ≤ 0.001).

**Conclusions:**

These findings suggest that people may face stigma across the cycle of losing and regaining weight after using a GLP-1, underscoring the need for stigma-reduction efforts in the context of weight management.

## An experimental investigation of the stigmatization of weight loss and regain from GLP-1 receptor agonist use and cessation

Glucagon-like peptide-1 receptor agonist medications, or GLP-1s, are a class of medications initially developed to treat diabetes. In 2021 and 2023, two GLP-1s, semaglutide and tirzepatide (a dual GLP-1/glucose-dependent insulinotropic polypeptide agonist), received FDA approval to treat obesity [1, 2], and they have since revolutionized the weight management field [3]. Evidence suggests that these new GLP-1s are effective for achieving clinically significant weight loss [4–6], although long-term follow-up studies are still needed [7]. Given the generally low, short-term efficacy of behavioral weight loss interventions [8–10] and the invasiveness of weight loss surgery [11], GLP-1s are an attractive treatment option for many higher-weight^1^ patients.

A key drawback of GLP-1s is that when people stop taking them (e.g., due to side effects, changes in insurance coverage), they tend to regain a substantial amount of weight within a year [12, 13]. Widespread weight regain resulting from GLP-1 cessation is concerning, both because weight cycling (i.e., losing and regaining weight) is physiologically taxing [14] and because people may be stigmatized for regaining weight. It is critical to understand and mitigate stigma associated with GLP-1 cessation and weight regain, as it has the potential to undermine people’s well-being and discourage them from seeking healthcare [15, 16].

These issues are pressing, as GLP-1 discontinuation and subsequent weight regain are poised to become increasingly common in the United States. Soon, many Americans may be forced to discontinue GLP-1s as they reach time- and cost-based limits of their health insurance coverage [17] and as access to compounded versions of GLP-1s is restricted [18]. To contextualize stigma directed at people who regain weight after GLP-1 cessation, it is also important to understand how people are regarded for losing weight with a GLP-1.

### Stigmatization of GLP-1 use and cessation

One narrative that has gained traction is that using GLP-1s to lose weight is “taking the easy way out” [19, 20], particularly as compared to behavioral weight loss methods. A similar narrative has been perpetuated about people who undergo bariatric (i.e., weight loss) surgery [21], a notion that is inherently stigmatizing, as it suggests that people who undergo surgery or use GLP-1s are lazy or undisciplined. Moreover, it reflects widespread bias against higher-weight people (i.e., weight stigma), which is unjust and harmful [15]. Indeed, there is a rich experimental literature about the stigma of having previously had obesity [22], with several studies finding that one’s method of weight loss (i.e., bariatric surgery vs. behavior change) can influence these judgments [23–26]. In general, this work finds that individuals who lose weight via bariatric surgery are regarded less highly than individuals who lose weight via behavior change. Some recent empirical work explored this phenomenon in the GLP-1 context [20], finding that a person who lost weight using a GLP-1 (i.e., a GLP-1 user) was evaluated more negatively than someone who lost weight via diet and exercise (i.e., a dieter). Consistent with the “taking the easy way out” narrative, this effect was mediated by the belief that using a GLP-1 was a “shortcut” to weight loss. Thus, GLP-1 users may be socially penalized. However, it is not clear how the magnitude of GLP-1-related stigma compares to more generalized stigma directed at higher-weight people. One potential benefit of losing weight might be reduced experiences of weight stigma, but this benefit may not be realized if people are stigmatized for doing so with a GLP-1.

Another important, underexplored aspect of GLP-1-related stigma is how GLP-1 users are regarded after discontinuing medication and regaining weight. Dieters who regain weight are judged harshly for “falling off the wagon” [27, 28], and similar judgments may be applied to GLP-1 users who regain weight. Moreover, GLP-1 users who regain weight may be accused of “squandering an opportunity” granted by these highly sought-after medications, similar to stigma directed at people who regain weight after bariatric surgery [29, 30]. To our knowledge, no empirical work has sought to understand the stigma of regaining weight after GLP-1 use.

GLP-1 users may also encounter judgment related to high medication costs. Insurance coverage for GLP-1s is limited, and out-of-pocket costs are extremely high (i.e., list prices ~$1200/month, or ~$700/month with manufacturer discounts) [31]. Thus, people may make assumptions about the socioeconomic status (SES) of individuals who use or discontinue GLP-1s. Indeed, some have pointed to the lack of GLP-1 affordability as a factor that could widen obesity-related health disparities [32, 33] and increase stigma at the intersection of class and body size [34]. For instance, if people perceive GLP-1 usage as a signal of high SES, someone who chooses not to use or discontinues the medication may be assumed to be unable to afford them, thereby layering classism on top of weight stigma. However, the more basic question of whether people make assumptions about SES based on GLP-1 use is not yet established.

### The present work

Across two pre-registered, randomized experiments, we investigated the stigma of weight loss and regain resulting from GLP-1 use and cessation. We asked participants to evaluate a fictional target and assessed two measures of weight stigma (i.e., endorsement of weight-related stereotypes about the target, willingness to affiliate with the target) and perceptions of the target’s SES.

As the literature in this field is nascent, Study 1 was designed to examine the stigmatization of losing weight using a GLP-1, both by replicating the few existing studies [20, 35] and by extending them by examining whether losing weight with a GLP-1 is more or less stigmatized than maintaining a higher weight. Therefore, we sought to compare how people evaluated someone who lost weight using a GLP-1 versus someone who either lost weight via diet and exercise or did not attempt weight loss. We anticipated that higher-weight people would be more stigmatized than people who lost weight using a GLP-1, so we hypothesized that the target who did not attempt weight loss would receive the most unfavorable evaluations, followed by the target who lost weight using a GLP-1. We expected the target who lost weight via diet and exercise to receive the most favorable evaluations.

Study 2 was designed to understand the stigmatization of weight regain after GLP-1 cessation. Participants evaluated a target who had either regained weight after discontinuing a GLP-1, regained weight after discontinuing a diet and exercise program, did not attempt weight loss, or lost weight and maintained it. Based on some unexpected findings in Study 1 (i.e., the GLP-1 weight loss target was evaluated more unfavorably than the no weight loss target; see *Study 1 Confirmatory Findings*), we hypothesized that the target who regained weight after discontinuing a GLP-1 would be evaluated most unfavorably, followed by the target who did not attempt weight loss, and then by the target who regained weight after discontinuing a diet and exercise program. We expected the target who lost weight and maintained it to receive the most favorable evaluations, as they were the only target who succeeded at losing weight.

In both studies, we tested the exploratory hypothesis that ratings of the target’s SES would be higher for the GLP-1 target than for the diet+exercise target. We also explored whether participants’ explicit or internalized weight bias moderated the effects of study condition on willingness to affiliate with the target. These moderation tests were pre-registered, but we did not specify directional hypotheses.

## Methods

### Participants

Participants were recruited from Prolific, an online survey platform, in October 2024 (Study 1; *N* = 607) and February 2025 (Study 2; *N* = 706). To be eligible, participants needed to be 18 years or older, fluent in English, and reside in the United States. Study 1 participants were excluded from Study 2, and participant-facing study descriptions did not mention weight, dieting, or GLP-1s. Recruitment targets were determined a priori based on power analyses conducted using G^*^Power Version 3[36];. For one-way ANOVA models with power of 0.9 and an anticipated small-to-medium effect size (*f* = 0.15), a minimum of 567 (Study 1) and 636 participants (Study 2) were needed. We set recruitment targets slightly above these minimums to ensure sufficient power for exploratory tests. For sample demographics and sample size by condition, see Tables 1–3.

**Table 1:** Demographic information.

*Demographic*	Mean [SD] or Frequency (%)	
	Study 1 (*N* = 607)	Study 2 (*N* = 706)
Age, in years	39.46 [12.99]	42.02 [13.39]
Self-Reported Gender Identity		
Man	239 (39.4%)	333 (47.2%)
Woman	355 (58.5%%)	359 (50.8%)
Transgender	9 (1.5%%)	4 (0.6%)
Non-Binary or Genderfluid	11 (1.8%)	6 (0.8%)
Not reported	4 (0.7%)	6 (0.8%)
Race/Ethnicity		
White	420 (69.2%)	559 (79.2%)
Hispanic or Latino/a/x	47 (7.7%)	51 (7.2%)
Asian or Pacific Islander	53 (8.7%)	45 (6.4%)
Black or African American	112 (18.5%)	79 (11.2%)
Native American	14 (2.3%)	11 (1.6%)
Middle Eastern or North African	0 (0%)	3 (0.4%)
Another race or ethnicity	2 (0.3%)	4 (0.6%)
Not reported	5 (0.8%)	8 (1.1%)
Annual Income		
$19,999 or less	70 (11.5%)	66 (9.3%)
$20,000 - $39,999	102 (16.8%)	98 (13.9%)
$40,000 - $59,999	129 (21.3%)	124 (17.6%)
$60,000 - $79,999	90 (14.8%)	94 (13.7%)
$80,000 - $99,999	65 (10.7%)	94 (13.7%)
$100,000 or more	141 (23.2%)	213 (30.2%)
Not reported	10 (1.6%)	12 (1.7%)
Education		
Less than a high school diploma	5 (0.8%)	5 (0.7%)
High school degree or equivalent	90 (14.8%)	98 (13.8%)
Some college, no degree	126 (20.8%)	116 (16.4%)
Associate degree	59 (9.7%)	87 (12.3%)
Bachelor’s degree	222 (36.6%)	268 (38%)
Master’s degree	78 (12.9%)	105 (14.9%)
Professional degree or doctorate	24 (4%)	22 (3.1%)
Not reported	3 (0.5%)	5 (0.7%)

For gender identity and race/ethnicity questions, participants were able to select all response options that applied, so percentages do not add up to 100.

**Table 2:** Study 1 descriptives and test statistics by condition.

	*Study Condition* Mean [SD]			ANOVA Test Statistics			
*Outcome*	GLP-1 Weight Loss (*n* = 203)	Diet + Exercise Weight Loss (*n* = 203)	No Weight Loss (*n* = 201)	df	*F*	*η*_*p*_^2^	*p*-value
**Weight-Related Stereotyping of Target**							
Positive Traits	4.59 [0.93]^a^	4.93 [0.86]^b^	4.88 [0.88]^b^	2, 604	8.66	0.03	≤ 0.001^***^
Negative Traits	2.63 [1.26]^b^	2.18 [0.92]^a^	2.45 [1.23]^ab^	2, 604	7.68	0.02	≤ 0.001^***^
Unhealthy	3.21 [1.52]^b^	2.59 [1.45]^a^	3.45 [1.65]^b^	2, 604	16.79	0.05	≤ 0.001^***^
**Willingness to Affiliate with Target**	4.99 [1.1]^a^	5.51 [0.92]^c^	5.25 [1.08]^b^	2, 603	12.54	0.04	≤ 0.001^***^

Matching superscript letters indicate that means are not significantly different from each other (*p* > 0.05) based on Tukey’s HSD tests, and they are ordered from lowest to highest significantly different mean value. Significance indicated by ^***^p ≤ 0.001.

**Table 3:** Study 2 descriptives and test statistics by condition.

	*Study Condition* Mean [SD]				ANOVA Test Statistics			
*Outcome*	GLP-1 Regain (*n* = 177)	Diet + Exercise Regain (*n* = 176)	No Weight Loss (*n* = 175)	Maintained Weight Loss (*n* = 178)	df	*F*	*η*_*p*_^2^	*p*-value
**Weight-Related Stereotyping of Target**								
Positive Traits	4.56 [0.97]^a^	4.66 [0.88]^ab^	4.85 [0.82]^b^	4.8 [0.91]^b^	3, 702	4	0.02	0.008^**^
Negative Traits	3.15 [1.38]^bc^	3.16 [1.24]^c^	2.82 [1.2]^b^	2.35 [1.13]^a^	3, 702	16.94	0.07	≤ 0.001^***^
Unhealthy	4.17 [1.72]^b^	3.94 [1.55]^b^	3.81 [1.6]^b^	2.83 [1.56]^a^	3, 702	23.89	0.09	≤ 0.001^***^
**Willingness to Affiliate with Target**	5.02 [1.1]^a^	5.11 [1.11]^ab^	5.24 [0.94]^ab^	5.33 [0.97]^b^	3, 702	3.51	0.01	0.02^*^

Matching superscript letters indicate that means are not significantly different from each other (*p* > 0.05) based on Tukey’s HSD tests, and they are ordered from lowest to highest significantly different mean value. Significance indicated by ^*^p ≤ 0.05, ^**^p ≤ 0.01, ^***^p ≤ 0.001.

### Procedures

We conducted two between-subjects randomized experiments. In both studies, participants were randomly assigned to a condition (via a randomizer embedded in the survey) and then read a brief description of a fictional target. Across all conditions, the target (age 38, no gender specified^2^, college-educated) was described as having had obesity since puberty, with a recent weight of around 220 pounds. These demographic details were designed after an average-aged, average-weight individual in the U.S. with a modal period of onset of obesity [39]. Further details about the target’s weight-related history varied by study condition.

In Study 1, we manipulated whether the target had lost weight (i.e., 35 pounds over the past year) by taking a GLP-1 medication (*GLP-1 weight loss* condition) or by going on a diet and exercising regularly (*diet+exercise weight loss* condition). In both weight loss conditions, we specified that the target was no longer in the “obese” BMI category to emphasize the results of the target’s weight loss efforts. In a third condition, the target description did not mention weight loss (*no weight loss* condition), indicating that the target currently had obesity.

In Study 2, we manipulated whether the target had lost weight with a GLP-1 medication and then regained it after stopping (*GLP-1 regain* condition), lost weight via diet and exercise and regained it after stopping (*diet+exercise regain* condition), never lost weight (*no weight loss* condition), or lost weight and maintained it, with the method of weight loss unspecified (*maintained weight loss* condition). In the *maintained weight loss* condition, the target was described as “no longer in the obese BMI category,” but for all other conditions, it was noted that the target had obesity.

Aside from condition-specific details, information about the target was identical across conditions (see *Supporting Information* for study manipulations). After reading the target description, participants completed questionnaires and received $1.60 ( ~ $15/hour).

### Measures

#### Weight-related stereotyping

On a scale from 1 (*not at all*) to 7 (*extremely*), participants rated the target on a variety of traits related to stereotypes of higher-weight people [40–42]. The positive traits were: honest, generous, sociable, productive, organized, friendly, outgoing, intelligent, warm, and humorous. The negative traits were: lazy, undisciplined, self-indulgent, unclean, weak-willed, sloppy, insecure, and sluggish. Scores for positive and negative traits were averaged to form reliable composite scales (Study 1:*α*_positive_ = 0.91_,_ *α*_negative_ = 0.92; Study 2:*α*_positive_ = 0.91_,_ *α*_negative_ = 0.92). We also assessed participants’ ratings of the trait “unhealthy,” which we analyzed individually because it is conceptually different from the other traits (i.e., about the target’s physical health rather than their personality or current state).

#### Willingness to affiliate

Participants reported their willingness to affiliate with the target using six items adapted from the Interpersonal Attraction Questionnaire ([43]; see [44] for a similar adaptation). An example items is: “I think I would enjoy [target’s] company.” Response options ranged from 1 (*strongly disagree*) to 7 (*strongly agree*), and scores were averaged (*α*_Study 1_ = 0.9_,_ *α*_Study 2_ = 0.89).

#### Explicit weight bias

Explicit weight bias was measured using the Empathy (7-item), Responsibility (6-item), and Socioeconomic Complexity (3-item) subscales of the Fat Attitudes Assessment Toolkit [45]. Subscale scores were averaged separately (*α*s > 0.89).

#### Internalized weight bias

Internalized weight bias was measured using the 11-item Weight Bias Internalization Scale-Modified [46]. Responses were averaged to create a composite score (both *α*s = 0.93).

#### Perceptions of the target’s SES

We measured perceptions of the target’s SES using the single-item MacArthur Scale of Subjective Social Status [47]. Participants viewed an image of a ladder and were told that the top represented people who were the best off in society (i.e., best jobs, most money), whereas the bottom represented people who were the worst off. They were asked to estimate where the target would fall on the ladder on a scale from 1 (*bottom rung*) to 10 (*top rung*).

### Analytic plan

#### Confirmatory analyses

Study 1 and Study 2 employed similar analytic approaches, and all analyses were conducted in RStudio 4.3.1. After computing composite scales, we confirmed that they approximated a normal distribution. We tested our confirmatory hypotheses by conducting one-way ANOVAs to determine whether key outcomes differed by study condition. Whenever an overall model was significant, we used Tukey’s Honestly Significant Difference (HSD) tests. We set *α* = 0.05, and due to randomization, we did not use any covariates. Groups did not differ by age, self-reported gender, race/ethnicity, income, or education (all *p*s > 0.06).

#### Exploratory analyses

We tested our moderation hypotheses by mean-centering each proposed moderator and testing the interaction between the moderator and study condition in a multiple linear regression model. For these tests, we focused on pairwise comparisons containing *GLP-1* conditions. To test our hypotheses about perceptions of the target’s SES, we used two-tailed independent samples t-tests to compare scores from participants in the *GLP-1* and *diet+exercise* conditions.

## Results

### Study 1

#### Confirmatory findings

Participants’ endorsement of weight-related stereotypes differed significantly by study condition (all *p*s < 0.001; see Table 2). Consistent with hypotheses, those seeing the *GLP-1 weight loss* target rated the individual as significantly lower in positive traits, higher in negative traits, and more unhealthy than those seeing the *diet+exercise weight loss* target did (Tukey HSD *p*s < 0.001). However, an unexpected pattern emerged for the *no weight loss* condition. We hypothesized that those seeing the *no weight loss* target would rate the individual less favorably than would those seeing either weight loss target. Instead, we found that they rated the target higher in positive traits and no different in negative traits or unhealthiness compared to the *GLP-1 weight loss* target (*p*_positive_ = 0.003; *p*_negative_ = 0.26; *p*_unhealthy_ = 0.26).

**Fig. 1:**
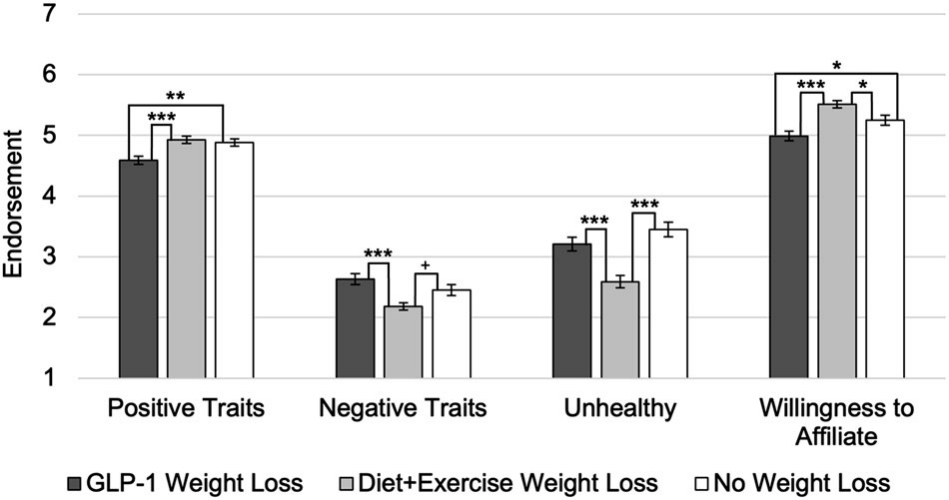
Key outcomes by condition for study 1. Mean endorsement of outcome measures by study condition. Error bars represent standard errors. Level of significance for follow-up Tukey’s HSD tests denoted by ***p ≤ 0.001, **p ≤ 0.01, *p ≤ 0.05, ^+^*p* ≤ 0.1.

Another unexpected pattern emerged for participants’ willingness to affiliate. Willingness differed significantly by condition (*F*(2, 603) = 12.54, *η*_*p*_^2^ = 0.04, *p* < 0.001), and all group means were significantly different from each other (*p*s < 0.04). Participants seeing the *diet+exercise weight loss* target were most willing to affiliate with the individual (*M* = 5.51, *SD* = 0.92), followed by those seeing the *no weight loss* target (*M* = 5.25, *SD* = 1.08), and then those seeing the *GLP-1 weight loss* target (*M* = 4.99, *SD* = 1.1). For a visual representation of these results, see Fig. 1.

#### Exploratory findings

There were no significant differences in ratings of the target’s SES between those viewing the *GLP-1 weight loss* individual (M = 5.95, SD = 1.27) and the diet+exercise weight loss individual (M = 5.82, SD = 1.42; *t*(399) = 0.99, *p* = 0.32). Moreover, effects were generally not moderated by participants’ levels of explicit or internalized weight bias (see Tables S1 and S2 and *Supporting Information*). However, two significant moderation effects emerged in models comparing the *GLP-1 weight loss* and *diet+exercise weight loss* conditions. Specifically, willingness to affiliate with the target was especially low among participants who viewed the GLP-1 weight loss target and had low empathy (*p* = 0.04) or high responsibility beliefs (p < 0.001), both of which represent greater weight bias.

### Study 2

#### Confirmatory findings

**Fig. 2:**
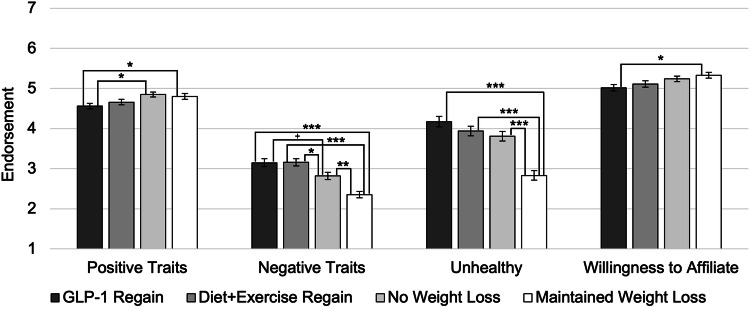
Key outcomes by condition for study 2. Mean endorsement of outcome measures by study condition. Error bars represent standard errors. Level of significance for follow-up Tukey’s HSD tests denoted by ***p ≤ 0.001, ** *p* ≤ 0.01, *p ≤ 0.05, ^+^*p* ≤ 0.1.

Weight-related stereotyping of the target differed significantly by study condition (*p*s < 0.008; see Table 3) but not as hypothesized (see Fig. 2). In general, we found that participants seeing the *GLP-1 regain* target and those seeing the *diet+exercise regain* target had similar responses; there were no differences in their ratings of the target’s positive traits, negative traits, or unhealthiness (Tukey’s HSD *p*s > 0.55). However, we observed differences between the two regain groups and the other groups. For instance, participants seeing the *no weight loss* target endorsed more positive traits for the individual than did those seeing the *GLP-1 regain* target (*p* = 0.01), but ratings of negative traits and unhealthiness were similar across the two groups (*p* > 0.06). In addition, those who viewed the *maintained weight loss* target rated the individual as significantly lower in negative traits (*p*s < 0.001) and less unhealthy (*p*s < 0.001) than did participants who viewed the *GLP-1 regain* target or *diet+exercise regain* target.

Participants’ willingness to affiliate with the target differed across groups (*F*(3, 702) = 3.51, *η*_*p*_^2^ = 0.01, *p* = 0.02). A follow-up test revealed that those seeing the *GLP-1 regain* target (*M* = 5.02, *SD* = 1.1) were significantly less willing to affiliate with the individual than those seeing the *maintained weight loss* target (*M* = 5.33, *SD* = 0.97).

#### Exploratory findings

Ratings of the target’s SES did not differ between those viewing the *GLP-1 regain* target *(M* = 5.75, *SD* = 1.5) and those viewing the *diet+exercise regain* target (*M* = 5.77, *SD* = 1.25; *t*(341) = –0.18, *p* = 0.85). Moreover, no relationships between study condition and willingness to affiliate were moderated by explicit or internalized weight bias (*p*s > 0.08; see Tables S3-S5).

## Discussion

The present work demonstrates that both losing weight with a GLP-1 *and* regaining weight after discontinuing a GLP-1 can result in stigma. In Study 1, we found that participants evaluated an individual who lost weight with a GLP-1 more harshly than an individual who lost weight via diet and exercise (i.e., higher in negative traits, lower in positive traits, more unhealthy, less willing to affiliate). Moreover, they evaluated an individual who lost weight with a GLP-1 even more harshly (i.e., lower in positive traits, less willing to affiliate) than a higher-weight individual who had not attempted weight loss. Study 2 tested whether regaining weight after discontinuing a GLP-1 is stigmatized, and hypotheses were partially confirmed. Participants stigmatized an individual who had regained weight after using a GLP-1, rating them less favorably than an individual who had lost weight and maintained it (i.e., higher in negative traits, lower in positive traits, more unhealthy, less willing to affiliate) and less favorably, again, than even an individual who had not attempted weight loss (i.e., lower in positive traits). However, the weight loss method prior to regain did not seem to influence stigma; there were no significant differences in weight-related stereotyping or willingness to affiliate across the GLP-1 and diet+exercise groups. Notably, the target who lost weight and maintained it was rated more favorably (i.e., higher in positive traits, lower in negative traits, less unhealthy) than all three of the targets who currently had obesity (i.e., targets from the *GLP-1 regain*, *diet+exercise regain*, and *no weight loss* conditions), suggesting that having a higher weight is harshly stigmatized regardless of a person’s prior weight loss attempts.

Our findings conceptually replicate the work of Post and Persky [20], who also found that participants evaluated a GLP-1 user more negatively than a dieter. We found that a similar pattern exists for people’s willingness to affiliate with someone who lost weight – participants reported greater interest in spending time with the dieter than the GLP-1 user. In addition, our findings extend the field’s understanding of the full cycle of GLP-1-related stigma by demonstrating that GLP-1 users and dieters face similar levels of stigma after regaining weight. Given that weight regain is the likely outcome of these weight loss methods [10, 12, 13], and that widespread weight regain after GLP-1 cessation may become increasingly common, it is crucial to understand the social consequences of weight regain.

Another key extension of this work is that it directly compares the stigma directed at GLP-1 users with the stigma directed at higher-weight people. Without this comparison, one cannot discern whether stigma directed at GLP-1 users represents a *decrease* from the status quo (i.e., lower than the amount of stigma endured at a higher weight). After significant weight loss, one might expect that a GLP-1 user could “escape” weight stigma. However, our findings suggest the opposite: that people may experience *more* stigma for losing weight with a GLP-1 than for remaining at a higher weight. These findings contrast those of a similar study of bariatric surgery stigma, which found that higher-weight targets without a stated weight loss history were stigmatized more (i.e., rated as less desirable mates) than normal-weight targets who had undergone bariatric surgery [26]. Moreover, the apparent inevitability of stigma for GLP-1 users is concerning, particularly given that a desire to escape weight stigma has been conceptualized as a driver of unhealthy weight control behaviors (e.g., purging, skipping meals) and social isolation [48, 49].

Finally, we found relatively little support for our exploratory hypotheses. We found some evidence of moderation by empathy and responsibility beliefs in Study 1, suggesting that people with high explicit weight bias may penalize GLP-1 users (vs. dieters) especially harshly. However, we did not find similar effects in Study 2, and we found no differences in perceptions of the target’s SES in either study. To avoid demand characteristics, we did not provide information about GLP-1 costs in the study manipulations, so our hypotheses relied on participants’ existing knowledge of GLP-1s. Thus, one explanation for the lack of differences observed in perceptions of the target’s SES is that the high costs of GLP-1s may not have been widely known among our participants. However, we also note that we did not specify whether the target had insurance coverage, so even if participants were familiar with the costs of GLP-1s, they may not have felt able to make SES-related judgments about the target.

### Strengths, limitations, and future directions

This work presents novel evidence about the stigma of weight loss and regain following GLP-1 use and cessation. It is the first study, to our knowledge, to investigate how stigma directed at GLP-1 users compares to stigma directed at higher-weight people, and it offers a timely examination of the stigma associated with regaining weight after discontinuing a GLP-1. Mapping the stigma GLP-1 users face across the weight loss/regain cycle is crucial to designing future bias-reduction efforts.

For both studies, we employed a randomized, between-subjects experiment in which participants were asked to evaluate a fictional target. Although this approach maximized internal validity and allowed for causal inference via precise study manipulations, it may have felt somewhat artificial to participants. To remedy this, future work could employ more immersive, realistic manipulations (e.g., lab-based interactions, virtual reality). Another limitation is that our study manipulations provided people with information they may not usually have when making social judgments. In everyday life, people only have access to others’ weight-related history if the information has been shared with them directly or if they make an assumption. Moreover, it is not possible to tell if a stranger has recently lost (or regained) weight after dieting or using a GLP-1. Therefore, it will be necessary for future work to examine GLP-1 users’ experiences of stigma in everyday life to quantify the frequency and intensity with which it is experienced. This work is also limited by the demographics of our participants, who were predominantly white, tended to be young, and had high income and educational attainment. To ensure that findings are generalizable, key next steps will be to replicate and extend this work with variations of the target (e.g., gender, race/ethnicity, prior attempts at weight loss, cost paid for medication) and diverse groups of participants, both in terms of participant demographics and preexisting knowledge of GLP-1s (e.g., general population, eligible patients, healthcare professionals). Finally, we note that many GLP-1 users also engage in lifestyle changes to lose weight. Future research should examine whether sharing about one’s concurrent lifestyle change efforts can buffer stigma faced by GLP-1 users.

## Conclusion

As GLP-1s have gained popularity, people have begun to stigmatize those who lose weight using GLP-1s. Moreover, people stigmatize those who have regained weight after discontinuing a GLP-1, demonstrating that GLP-1 users may find it difficult to escape weight stigma and its deleterious social, health, and economic consequences [50]. Thus, individuals pursuing these treatments can become mired in a “damned if you do, damned if you don’t” situation, wherein remaining at a higher weight means enduring weight stigma, but losing (and potentially regaining) weight after using a GLP-1 also makes them vulnerable to judgment and discrimination. There is, therefore, a clear need for interventions that address the stigma associated with using GLP-1s for weight loss. Furthermore, it will be crucial for stigma-reduction efforts to be wide-reaching and designed for a variety of audiences (e.g., public health campaigns for the general population, specialized training for healthcare professionals, educational materials for patients to combat self-stigma), particularly as GLP-1s continue to grow in popularity.

## Supplementary information

Supplementary Information

## Data Availability

Materials used to conduct this work (e.g., study manipulations, measures) and de-identified datasets are available in a public archive: 10.17605/OSF.IO/7FM2R.

## Footnotes

1 We use the term “higher-weight” to refer to people with obesity (i.e., body mass index (BMI) ≥ 30 kg/m^2^).

2 Gender was made ambiguous because prior work suggests that the nature of weight stigmatization may differ by gender [37, 38].
