# Supplementary material for: An experimental investigation of the stigmatization of weight loss and regain from GLP-1 receptor agonist use and cessation
Source: Int J Obes (Lond). 2026 Apr 3;50(7):1478–85. doi: 10.1038/s41366-026-02061-y (PMC13391369; doi:10.1038/s41366-026-02061-y)
Supplement: Supplementary file 1 — Supplementary Information [file 41366_2026_2061_MOESM1_ESM.docx]

**Supporting Information**

**Article Title: An experimental investigation of the stigmatization of weight loss and regain from GLP-1 receptor agonist use and cessation**

**Authors:** Erin C. Standen, Sean M. Phelan, & A. Janet Tomiyama

**Study 1 Manipulation**

[All Conditions]

Please read the following description. We are curious about your first impressions of the person described in the text.

Jamie is a 38-year-old who lives in the United States. After earning an undergraduate degree in Business Administration, Jamie began a job as a salesperson and has worked on corporate sales and marketing teams ever since. Outside of work, Jamie enjoys listening to music, taking dog walks, and spending time with friends and family. Since puberty, Jamie has had obesity, meaning having had a body mass index (BMI) of 30 or greater. In recent years, Jamie’s weight has stayed around 220 pounds.

[Condition-Specific Portion]

GLP-1 Weight Loss Condition

- Over the past year, Jamie lost 35 pounds using weekly injections of semaglutide (e.g., “Ozempic” or “Wegovy”), a prescription medication. Now, Jamie weighs 185 pounds and is no longer in the “obese” BMI category.

Diet + Exercise Weight Loss Condition

- Over the past year, Jamie lost 35 pounds by going on a diet and exercising several times per week. Now, Jamie weighs 185 pounds and is no longer in the “obese” BMI category.

No Weight Loss (i.e., Control) Condition

- N/A – description ends

**Study 2 Manipulation**

[All Conditions]

Please read the following description. We are curious about your first impressions of the person described in the text.

Jamie is a 38-year-old who lives in the United States. After earning an undergraduate degree in Business Administration, Jamie began a job as a salesperson and has worked on corporate sales and marketing teams ever since. Outside of work, Jamie enjoys listening to music, taking dog walks, and spending time with friends and family. Since puberty, Jamie has had obesity, meaning having had a body mass index (BMI) of 30 or greater. In recent years, Jamie’s weight has stayed around 220 pounds.

[Condition-Specific Portion]

GLP-1 Regain Condition

- Last year, Jamie lost 35 pounds using weekly injections of semaglutide (e.g., “Ozempic” or “Wegovy”), a prescription medication. At one point, Jamie’s weight was down to 185 pounds, which meant Jamie was no longer in the “obese” BMI category. However, after stopping the medication, Jamie has now regained the weight and is back to 220 pounds.

Diet + Exercise Regain Condition

- Last year, Jamie lost 35 pounds by going on a diet and exercising several times per week. At one point, Jamie’s weight was down to 185 pounds, which meant Jamie was no longer in the “obese” BMI category. However, after stopping the diet and exercise plan, Jamie has now regained the weight and is back to 220 pounds.

No Weight Loss Condition

- N/A - vignette ends

Lost Weight and Maintained Condition

- Last year, Jamie lost 35 pounds. Now, Jamie weighs 185 pounds and is no longer in the “obese” BMI category.

**Study 1: Exploratory Moderation Findings**

For the most part, effects were not moderated by participants’ levels of explicit weight bias or internalized weight bias (see Tables S1 and S2). However, two significant moderation effects emerged in models comparing the *GLP-1 weight loss* and *diet+exercise weight loss* conditions. We found that empathy for higher-weight people moderated the relationship between study condition and willingness to affiliate with the target (*b* = 0.19, SE *b* = 0.09, *β* = 0.14, *p* = 0.04). Participants seeing the *GLP-1 weight loss* target with low empathy (i.e., more explicit weight bias) were especially low in willingness to affiliate with the target. Similarly, the relationship between study condition and willingness to affiliate was moderated by participants’ ratings of higher-weight people’s responsibility for their weight (*b* = -0.27, SE *b* = 0.08, *β* = -0.22, *p* < 0.001). Those viewing the *GLP-1 weight loss* target who were high in responsibility (i.e., more explicit weight bias) reported especially low willingness to affiliate with the target.

These two significant moderation findings suggest that people who are high in explicit weight bias may socially penalize GLP-1 users (vs. dieters) to a greater extent than people who are lower in explicit weight bias. However, it is important to note that this effect did not emerge for all aspects of weight bias that we measured (e.g., did not emerge for socioeconomic or internalized weight bias), nor did it emerge when comparing the *GLP-1 weight loss* and *no weight loss* conditions. Finally, these moderation findings did not emerge in Study 2 when examining the stigma associated with weight regain.

**Table S1.** *Exploratory Tests of Moderation for* GLP-1 Weight Loss *vs.* Diet + Exercise Weight Loss *Conditions in Study 1*

|  | **Outcome: Willingness to Affiliate with Target** | | | |
| --- | --- | --- | --- | --- |
|  | ***b*** | **SE *b*** | ***β* [95% CI]** | ***p*-value** |
| ***Moderator: Explicit weight bias - Empathy*** |  |  |  |  |
| Condition: *GLP-1 weight loss* | -0.52 | 0.07 | -0.25 [-0.71, -0.32] | < 0.001^***^ |
| Empathy | 0.1 | 0.07 | 0.1 [-0.03, 0.23] | 0.13 |
| **Condition*Empathy** | **0.19** | **0.09** | **0.14 [0.01, 0.38]** | **0.04**^*^ |
| Overall Model Statistics: | *F*(3, 401) = 16.91, *R*^2^ = 0.11, *p* < 0.001^***^ | | | |
| ***Moderator: Explicit weight bias - Responsibility*** |  |  |  |  |
| Condition: *GLP-1 weight loss* | -0.5 | 0.1 | -0.24 [-0.69, -0.31] | < 0.001^***^ |
| Responsibility | -0.1 | 0.05 | -0.12 [-0.2, 0] | 0.05^+^ |
| **Condition*Responsibility** | **-0.27** | **0.08** | **-0.22 [-0.41, -0.12]** | **< 0.001^***^** |
| Overall Model Statistics: | *F*(3, 401) = 25.53, *R*^2^ = 0.15, *p* < 0.001^***^ | | | |
| ***Moderator: Explicit weight bias - Socioeconomic*** |  |  |  |  |
| Condition: *GLP-1 weight loss* | -0.52 | 0.1 | -0.25 [-0.72, -0.32] | < 0.001^***^ |
| Socioeconomic | 0.04 | 0.05 | 0.05 [-0.06, 0.13] | 0.46 |
| Condition*Socioeconomic | 0.06 | 0.07 | 0.06 [-0.07, 0.2] | 0.35 |
| Overall Model Statistics: | *F*(3, 401) = 10.45, *R*^2^ = 0.07, *p* < 0.001^***^ | | | |
| ***Moderator: Internalized Weight Bias*** |  |  |  |  |
| Condition: *GLP-1 weight loss* | -0.51 | 0.1 | -0.24 [-0.71, -0.31] | < 0.001^***^ |
| Internalized Weight Bias | 0.07 | 0.05 | 0.1 [-0.03, 0.16] | 0.16 |
| Condition*Internalized Weight Bias | 0.05 | 0.07 | 0.05 [-0.08, 0.18] | 0.46 |
| Overall Model Statistics: | *F*(3, 401) = 11.63, *R*^2^ = 0.07, *p* < 0.001^***^ | | | |

*Note.* Significant moderation effects are shown in bold. The *diet + exercise weight loss* condition was used as the reference group. For the Empathy and Socioeconomic measures, higher scores represent lower bias. For the Responsibility and Internalized weight bias measures, higher scores represent higher bias. Significance indicated by ^+^*p* ≤ 0.10, ^*^*p* ≤ 0.05,^***^*p* ≤ 0.001.

**Table S2.** *Exploratory Tests of Moderation for* GLP-1 Weight Loss *vs.* No Weight Loss *Conditions in Study 1*

|  | **Outcome: Willingness to Affiliate with Target** | | | |
| --- | --- | --- | --- | --- |
|  | ***b*** | **SE *b*** | ***β* [95% CI]** | ***p* -value** |
| ***Moderator: Explicit weight bias - Empathy*** |  |  |  |  |
| Condition: *GLP-1 weight loss* | -0.3 | 0.11 | -0.13 [-0.5, -0.09] | 0.006^**^ |
| Empathy | 0.22 | 0.06 | 0.22 [0.08, 0.34] | < 0.001^***^ |
| Condition*Empathy | 0.08 | 0.09 | 0.05 [-0.1, 0.26] | 0.41 |
| Overall Model Statistics: | *F*(3, 397) = 11.84, *R*^2^ = 0.08, *p* < 0.001^***^ | | | |
| ***Moderator: Explicit weight bias - Responsibility*** |  |  |  |  |
| Condition: *GLP-1 weight loss* | -0.22 | 0.1 | -0.1 [-0.42, -0.02] | 0.03^*^ |
| Responsibility | -0.22 | 0.06 | -0.25 [-0.34, -0.11] | < 0.001^***^ |
| Condition*Responsibility | -0.14 | 0.08 | -0.11 [-0.31, 0.02] | 0.09^+^ |
| Overall Model Statistics: | *F*(3, 397) = 19.58, *R*^2^ = 0.12, *p* < 0.001^***^ | | | |
| ***Moderator: Explicit weight bias - Socioeconomic*** |  |  |  |  |
| Condition: *GLP-1 weight loss* | -0.28 | 0.11 | -0.13 [-0.49, -0.06] | 0.01^*^ |
| Socioeconomic | 0.09 | 0.05 | 0.12 [-0.01, 0.18] | 0.08^+^ |
| Condition*Socioeconomic | 0.01 | 0.07 | 0.01 [-0.13, 0.16] | 0.85 |
| Overall Model Statistics: | *F*(3, 397) = 4.14, *R*^2^ = 0.02, *p* = 0.007^**^ | | | |
| ***Moderator: Internalized Weight Bias*** |  |  |  |  |
| Condition: *GLP-1 weight loss* | -0.26 | 0.11 | -0.12 [-0.48, -0.05] | 0.02^*^ |
| Internalized Weight Bias | 0.04 | 0.06 | 0.06 [-0.07, 0.15] | 0.44 |
| Condition*Internalized Weight Bias | 0.07 | 0.08 | 0.07 [-0.08, 0.22] | 0.34 |
| Overall Model Statistics: | *F*(3, 397) = 3.85, *R*^2^ = 0.02, *p* = 0.009^**^ | | | |

*Note.* The *no weight loss* condition was used as the reference group. For the Empathy and Socioeconomic measures, higher scores represent lower bias. For the Responsibility and Internalized weight bias measures, higher scores represent higher bias. Significance indicated by ^+^*p* ≤ 0.10, ^*^*p* ≤ 0.05, ^**^*p* ≤ 0.01, ^***^*p* ≤ 0.001.

**Table S3.** *Exploratory Tests of Moderation for* GLP-1 Regain *vs.* Diet + Exercise Regain *Conditions in Study 2*

|  | **Outcome: Willingness to Affiliate with Target** | | | |
| --- | --- | --- | --- | --- |
|  | ***b*** | **SE *b*** | ***β* [95% CI]** | ***p* -value** |
| ***Moderator: Explicit weight bias - Empathy*** |  |  |  |  |
| Condition: *GLP-1 regain* | -0.10 | 0.11 | -0.04 [-0.31, 0.12] | 0.39 |
| Empathy | 0.39 | 0.07 | 0.4 [0.25, 0.53] | < 0.001^***^ |
| **Condition*Empathy** | -0.11 | 0.10 | -0.08 [-0.3, 0.08] | 0.27 |
| Overall Model Statistics: | *F*(3, 348) = 16.22, *R*^2^ = 0.12, *p* < 0.001^***^ | | | |
| ***Moderator: Explicit weight bias - Responsibility*** |  |  |  |  |
| Condition: *GLP-1 regain* | -0.12 | 0.11 | -0.05 [-0.34, 0.1] | 0.29 |
| Responsibility | -0.35 | 0.07 | -0.38 [-0.49, -0.21] | < 0.001^***^ |
| **Condition*Responsibility** | 0.04 | 0.09 | 0.03 [-0.14, 0.22] | 0.69 |
| Overall Model Statistics: | *F*(3, 348) = 17.3, *R*^2^ = 0.12, *p* < 0.001^***^ | | | |
| ***Moderator: Explicit weight bias - Socioeconomic*** |  |  |  |  |
| Condition: *GLP-1 regain* | -0.10 | 0.11 | -0.04 [-0.32, 0.13] | 0.4 |
| Socioeconomic | 0.18 | 0.05 | 0.26 [0.08, 0.28] | < 0.001^***^ |
| **Condition*Socioeconomic** | -0.03 | 0.07 | -0.03 [-0.17, 0.11] | 0.65 |
| Overall Model Statistics: | *F*(3, 348) = 7.19, *R*^2^ = 0.05, *p* < 0.001^***^ | | | |
| ***Moderator: Internalized Weight Bias*** |  |  |  |  |
| Condition: *GLP-1 regain* | -0.10 | 0.12 | -0.04 [ -0.33, 0.13] | 0.41 |
| Internalized Weight Bias | 0.10 | 0.06 | 0.12 [-0.02, 0.21] | 0.11 |
| **Condition*Internalized Weight Bias** | -0.08 | 0.08 | -0.07 [-0.24, 0.09] | 0.35 |
| Overall Model Statistics: | *F*(3, 348) = 1.1, *R*^2^ < 0.001, *p* = 0.35 | | | |

*Note.* The *diet + exercise regain* condition was used as the reference group. For the Empathy and Socioeconomic measures, higher scores represent lower bias. For the Responsibility and Internalized weight bias measures, higher scores represent higher bias. Significance indicated by ^***^*p* ≤ 0.001.

**Table S4.** *Exploratory Tests of Moderation for* GLP-1 Regain *vs.* No Weight Loss *Conditions in Study 2*

|  | **Outcome: Willingness to Affiliate with Target** | | | |
| --- | --- | --- | --- | --- |
|  | ***b*** | **SE *b*** | ***β* [95% CI]** | ***p* -value** |
| ***Moderator: Explicit weight bias - Empathy*** |  |  |  |  |
| Condition: *GLP-1 regain* | -0.21 | 0.11 | -0.10 [-0.41, 0] | 0.05^+^ |
| Empathy | 0.24 | 0.07 | 0.25 [0.1, 0.37] | < 0.001^***^ |
| **Condition*Empathy** | 0.05 | 0.10 | 0.04 [-0.14, 0.23] | 0.63 |
| Overall Model Statistics: | *F*(3, 345) = 11.48, *R*^2^ = 0.08, *p* < 0.001^***^ | | | |
| ***Moderator: Explicit weight bias - Responsibility*** |  |  |  |  |
| Condition: *GLP-1 regain* | -0.16 | 0.10 | -0.08 [-0.36, 0.05] | 0.13 |
| Responsibility | -0.19 | 0.06 | -0.24 [-0.32, -0.07] | < 0.001^***^ |
| **Condition*Responsibility** | -0.12 | 0.08 | -0.10 [ -0.28, 0.05] | 0.17 |
| Overall Model Statistics: | *F*(3, 345) = 14.63, *R*^2^ = 0.11, *p* < 0.001^***^ | | | |
| ***Moderator: Explicit weight bias - Socioeconomic*** |  |  |  |  |
| Condition: *GLP-1 regain* | -0.22 | 0.11 | -0.11 [-0.44, -0.01] | 0.04^*^ |
| Socioeconomic | 0.02 | 0.05 | 0.04 [-0.08, 0.12] | 0.65 |
| **Condition*Socioeconomic** | 0.12 | 0.07 | 0.14 [-0.01, 0.26] | 0.08^+^ |
| Overall Model Statistics: | *F*(3, 345) = 4.59, *R*^2^ = 0.03, *p* = 0.004^**^ | | | |
| ***Moderator: Internalized Weight Bias*** |  |  |  |  |
| Condition: *GLP-1 regain* | -0.23 | 0.11 | -0.11 [-0.44, -0.01] | 0.04^*^ |
| Internalized Weight Bias | -0.07 | 0.06 | -0.09 [-0.18, 0.04] | 0.22 |
| **Condition*Internalized Weight Bias** | 0.09 | 0.08 | 0.09 [-0.07, 0.24] | 0.27 |
| Overall Model Statistics: | *F*(3, 345) = 1.89, *R*^2^ = 0.01, *p* = 0.13 | | | |

*Note.* The *no weight loss* condition was used as the reference group. For the Empathy and Socioeconomic measures, higher scores represent lower bias. For the Responsibility and Internalized weight bias measures, higher scores represent higher bias. Significance indicated by ^+^*p* ≤ 0.10, ^*^*p* ≤ 0.05, ^**^*p* ≤ 0.01, ^***^*p* ≤ 0.001.

**Table S5.** *Exploratory Tests of Moderation for* GLP-1 Weight Loss *vs.* Maintained Weight Loss *Conditions in Study 2*

|  | **Outcome: Willingness to Affiliate with Target** | | | |
| --- | --- | --- | --- | --- |
|  | ***b*** | **SE *b*** | ***β* [95% CI]** | ***p* -value** |
| ***Moderator: Explicit weight bias - Empathy*** |  |  |  |  |
| Condition: *GLP-1 weight loss* | -0.29 | 0.11 | -0.14 [-0.49, -0.08] | 0.01^*^ |
| Empathy | 0.32 | 0.07 | 0.33 [0.18, 0.46] | < 0.001^***^ |
| **Condition*Empathy** | -0.04 | 0.10 | -0.03 [-0.23, 0.15] | 0.68 |
| Overall Model Statistics: | *F*(3, 350) = 15.85, *R*^2^ = 0.11, *p* < 0.001^***^ | | | |
| ***Moderator: Explicit weight bias - Responsibility*** |  |  |  |  |
| Condition: *GLP-1 weight loss* | -0.28 | 0.11 | -0.13 [-0.49, -0.07] | 0.01^*^ |
| Responsibility | -0.18 | 0.06 | -0.22 [-0.31, -0.06] | < 0.001^***^ |
| **Condition*Responsibility** | -0.13 | 0.09 | -0.11 [-0.3, 0.04] | 0.14 |
| Overall Model Statistics: | *F*(3, 350) = 15.39, *R*^2^ = 0.11, *p* < 0.001^***^ | | | |
| ***Moderator: Explicit weight bias - Socioeconomic*** |  |  |  |  |
| Condition: *GLP-1 weight loss* | -0.32 | 0.11 | -0.15 [-0.54, -0.11] | < 0.001^***^ |
| Socioeconomic | 0.09 | 0.05 | 0.13 [-0.01, 0.19] | 0.09^+^ |
| **Condition*Socioeconomic** | 0.06 | 0.07 | 0.06 [-0.08, 0.2] | 0.43 |
| Overall Model Statistics: | *F*(3, 350) = 6.92, *R*^2^ = 0.05, *p* < 0.001^***^ | | | |
| ***Moderator: Internalized Weight Bias*** |  |  |  |  |
| Condition: *GLP-1 weight loss* | -0.33 | 0.11 | -0.16 [-0.55, -0.11] | < 0.001^***^ |
| Internalized Weight Bias | -0.01 | 0.05 | -0.02 [-0.12, 0.09] | 0.8 |
| **Condition*Internalized Weight Bias** | 0.03 | 0.08 | 0.03 [-0.12, 0.18] | 0.68 |
| Overall Model Statistics: | *F*(3, 349) = 3.04, *R*^2^ = 0.02, *p* = 0.03^*^ | | | |

*Note.* The *maintained weight loss* condition was used as the reference group. For the Empathy and Socioeconomic measures, higher scores represent lower bias. For the Responsibility and Internalized weight bias measures, higher scores represent higher bias. Significance indicated by ^*^*p* ≤ 0.05, ^**^*p* ≤ 0.01, ^***^*p* ≤ 0.001.
